# Revealing the Role of Zinc Ions in Atherosclerosis Therapy via an Engineered Three‐Dimensional Pathological Model

**DOI:** 10.1002/advs.202300475

**Published:** 2023-04-24

**Authors:** Ying Wang, Nan Huang, Zhilu Yang

**Affiliations:** ^1^ Dongguan Key Laboratory of Smart Biomaterials and Regenerative Medicine The Tenth Affiliated Hospital of Southern Medical University Dongguan 523059 P. R. China; ^2^ Guangdong Provincial Key Laboratory of Cardiac Function and Microcirculation Guangzhou 510080 P. R. China; ^3^ Department of Cardiology Third People's Hospital of Chengdu Affiliated to Southwest Jiaotong University Chengdu 610031 P. R. China

**Keywords:** atherosclerosis models, biodegradable vascular stents, layered arterial walls, zinc ions evaluation, zinc‐based alloys

## Abstract

An incomplete understanding of the cellular functions and underlying mechanisms of zinc ions released from zinc‐based stents in atherosclerosis (AS) therapy is one of the major obstacles to their clinical translation. The existing evaluation methodology using cell monolayers has limitations on accurate results due to the lack of vascular architectures and pathological features. Herein, the authors propose a 3D biomimetic AS model based on a multi‐layer vascular structure comprising endothelial cells and smooth muscle cells with hyperlipidemic surroundings and inflammatory stimulations as AS‐prone biochemical conditions to explore the biological functions of zinc ions in AS therapy. Concentration‐dependent biphasic effects of zinc ions on cell growth are observed both in cell monolayers and 3D AS models. Nevertheless, the cells within 3D AS model exhibit more accurate biological assessments of the zinc ions, as evidenced by augmented pathological features and significantly higher half‐maximal inhibitory concentration values against zinc ions. Based on such a developed 3D biomimetic AS model, the inhibitory effects on the deoxyribonucleic acid (DNA) synthesis, significantly influenced biological processes like cell motility, proliferation, and adhesion, and several potential bio‐targets of zinc ions of cells are revealed.

## Graphical abstract

The authors propose a 3D atherosclerosis (AS) model in vitro to investigate the cellular functions and underlying mechanisms of zinc ions. The cells within the 3D AS model exhibit more accurate biological assessments of the zinc ions, as evidenced by augmented pathological features, significantly higher half‐maximal inhibitory concentration values against zinc ions, and revealed biological processes and potential bio‐targets.

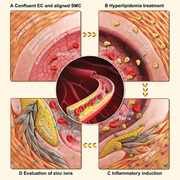

## 1 Introduction

Cardiovascular diseases (CVDs) have been the leading cause of death over the past few decades. Atherosclerosis (AS), contributing to the formation of fatty lesions and vascular occlusions on the arterial wall, is the major pathological basis of CVDs.^[^ ^1^ ^]^ To date, the implantation of vascular stents is the dominant strategy and has been used for more than 30 years to treat vascular stenosis and occlusion as well as extend the life expectancy of patients with AS.^[^ ^2^ ^]^ And bioabsorbable metals promote a trend toward implantable biodegradable vascular stents.^[^ ^3^ ^]^ Among others, zinc‐based alloys recently emerged as promising implantable stent materials due to the physiological role of zinc and the favorable degradation behavior.

Zinc ions (II), the dominant degradation product of zinc, gradually release into the surrounding tissue and influence numerous cellular activities.^[^ ^4^ ^]^ For example, zinc ions released from zinc oxide nanoparticles suppress vasculogenesis in human endothelial colony‐forming cells.^[^ ^4a^ ^]^ However, the controlled release of zinc ions from ZnP‐coated zinc metal at a concentration below 40 µg mL^−1^ significantly increased cell viability and adhesion of endothelial cells (ECs) exposed to extract media.^[^ ^5^ ^]^ In another example, low tissue levels of zinc ions were associated with increased disease measured in people with established AS.^[^ ^4b^ ^]^ Although a concentration dependency of physiological cellular functions on zinc ions has been revealed, there is almost no detailed analysis of zinc ions in the pathological ambiance of AS,^[^ ^4c^ ^]^ which places a major burden on the acceptance of zinc for implantation. Thus, it is crucial to consider the significantly different cellular behavior of smooth muscle cells (SMCs) and ECs at the pathological focus compared with healthy tissue for comprehending the AS progression as well as the potential mechanism reactions of zinc ions.^[^ ^6^ ^]^

Endothelial dysfunction caused by EC damage and the excessive formation of extracellular matrix (ECM) induced by abnormal proliferation of SMCs is a critical step of AS occurrence.^[^ ^7^ ^]^ In this regard, the employment of pathological conditions in vitro analytical model systems can provide an accurate understanding of the fundamental mechanisms of zinc ions on endothelial proliferation, vascular remodeling, and inflammatory responses.^[^ ^4c^ ^]^ In turn, the obtained comprehensive mechanism reactions further guide the optimization of the degradation behavior of zinc‐based bioabsorbable materials.^[^ ^3^ ^]^ Accordingly, more sophisticated efforts are urgently in need to comprehend the cellular functions and potential mechanisms of zinc ions within the pathological tissues or disease models in vitro, thereby promoting the application of zinc‐based bioabsorbable materials in vascular stents.

To date, hydrogels have been established as an effective tool in constructing well‐defined architectures with the precise spatial arrangement of multiple cell types and recreating physiological environments in vitro.^[^ ^8^ ^]^ In such cases, on the one hand, progress in developing vascular analogs has been reported using hydrogels incorporating ECs and SMCs with spatial separation, mimicking multi‐layered arterial walls.^[^ ^9^ ^]^ Among others, gelatin methacrylate (GelMA) hydrogel, derived from gelatin, has gained great popularity in tissue engineering strategy due to the good biocompatibility and tunable mechanical properties, providing an excellent physiological environment for cells compared to the traditional 2D cell culture approach.^[^ ^10^ ^]^ On the other hand, hydrogel matrixes can carry various cytokines, providing favorable physiological conditions.^[^ ^11^ ^]^ Basically, hyperlipidemic surroundings and inflammatory factors are key inducers in the formation of AS.^[^ ^6^, ^12^ ^]^ It was reported that oxidized low‐density lipoprotein cholesterol (ox‐LDL) exerts a plethora of effects to promote EC apoptosis, plaque progression, and inflammatory interactions between monocytes and the underlying vessel wall.^[^ ^7c^ ^]^ Besides, arterial SMCs internalize ox‐LDL, forming foam cells and being prone to lead to a necrotic core in the atherosclerotic plaque.^[^ ^1^ ^]^ In such cases, SMCs transform from the contractile phenotype to the synthetic phenotype, migrating toward the atherosclerotic plaque and secreting ECM molecules.^[^ ^7b^ ^]^ Additionally, AS is characterized by chronic inflammation.^[^ ^13^ ^]^ Several risk factors, such as tumor necrosis factor‐*α* (TNF‐*α*) and interleukin‐1*β* (IL‐1*β*), provoke inflammation, resulting in disordered lipid metabolism and damage of the endothelium.^[^ ^14^ ^]^

**Figure 1:**
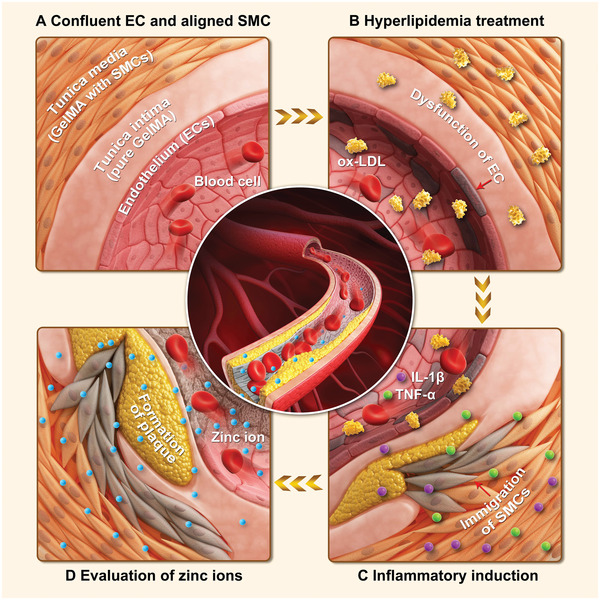
Overview of the study. Schematic illustration of the 3D AS model based on the layered arterial walls using GelMA hydrogel to investigate the molecular mechanisms and cellular reactions toward zinc ions at various concentrations. A) The physiologic paradigm of the arterial wall comprised of the tunica intima and tunica media. B) Artificial arterial wall comprised of the tunica intima and tunica media, formed by an EC‐layer and pure GelMA hydrogel, GelMA hydrogel encapsulating SMCs, respectively. Two key inducers of AS, hyperlipidemic environment and C) inflammatory cytokines, determined AS‐prone biochemical conditions. D) The engineered 3D AS model is employed to investigate the cellular effects and mechanistic molecular reactions of zinc ions at various concentrations.

Hence, we propose a 3D AS model that incorporates specific pathological features to investigate the cellular functions and underlying mechanisms of zinc ions at various concentrations (**Figure** **1**). As depicted in Figure 1A, the formation of a normal arterial wall with a layered architecture comprising various cell types was initially explored using GelMA hydrogel. Subsequently, hyperlipidemic surroundings and inflammatory stimulations were introduced as two key inducers stimulating AS progression as well as engineering the 3D AS model (Figure 1B). The pathological features of ECs and SMCs were detected by altered SMC phenotype, dysfunction of endothelium, and bioefficacy assessments of the constructed AS model after the treatment of ox‐LDL, TNF‐*α*, and IL‐1*β* along with the 2D cultured cells as comparisons. Further, we analyzed the cell response by cell proliferation and cell cycle, underlying mechanisms of zinc ions involved in AS by Gene Ontology (GO) functional analysis, as well as several potential targets based on the Kyoto Encyclopedia of Genes and Genomes (KEGG) pathway comparison using this engineered 3D AS model (Figure 1C).

## 2 Results and Discussions

### 2.1 Formation of 3D Highly Biomimetic Arterial Wall Model and Biological Performance of Cells

**Figure 2:**
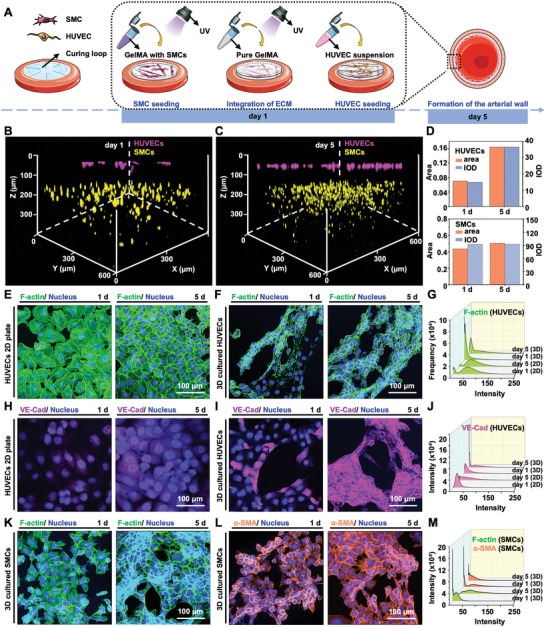
Formation of the healthy 3D arterial wall model and biological performance of HUVECs and SMCs. A) Schematic illustration of the normal 3D arterial wall model based on two‐layered GelMA hydrogel with HUVECs and SMCs in spatial separation and its experimental timeline. B,C) Fluorescence microscopy images showing the distribution of HUVECs (in purple) and SMCs (in yellow) within the constructed arterial models, and D) measurement of fluorescence area and IOD of HUVECs and SMCs for various time points (1 and 5 days), respectively. E,F) Fluorescence microscopy images illustrating the coalignment of actin filament of HUVECs in the 2D plate culture and on the surface of GelMA, respectively, and G) the measurement of the fluorescence intensity profile of HUVEC‐actin. H,I) CLSM images showing the endothelial‐specific junction protein of HUVECs both in the 2D plate culture and surface of GelMA (1 and 5 days), respectively, and J) the measurement of the fluorescence intensity profile of VE‐cad of HUVECs. K,L) Fluorescence microscopy images illustrating the coalignment of actin filament and *α*‐SMA expression of SMCs within GelMA (1 and 5 days), respectively, and M) the measurements of the fluorescence intensity profile of actin and *α*‐SMA of SMCs.

The native arterial wall comprises, from inside to outside, tunica intima, consisting of endothelium and subendothelial ECM, and tunica media.^[^ ^12a^ ^]^ The endothelium is a single layer of ECs, residing on the basement membrane that provides mechanical support as well as space for nutrient diffusion.^[^ ^15^ ^]^ The tunica media consists of quiescent SMCs embedded in a well‐established ECM, guiding the contraction and dilatation of normal vasculature.^[^ ^7a^ ^]^ Considering the integral structure of the artery, we developed a 3D biomimetic arterial wall model based on two‐layered GelMA hydrogel with human umbilical vein endothelial cells (HUVECs) and SMCs in spatial separation, as described below (**Figure** **2**A). Initially, GelMA with SMCs suspension was directly fixed to produce a tunica media with the appropriate ECM matrix. Subsequently, to establish subendothelial ECM patterning, GelMA without cells was cross‐linked on the adjacent tunica media as a membrane for HUVEC adhesion and proliferation. Finally, HUVECs were employed to decorate the surface of blank GelMA to simulate the endothelium, thus leading to the formation of arterial wall analogs.

The HUVECs and SMCs within the fabricated arterial wall analogs were visualized using a confocal laser scanning microscope (CLSM, LSM 900, Zeiss, Jena, Germany). We labeled HUVECs (in purple) and SMCs (in yellow) with distinct cell trackers to observe the cell distribution and growth during the 5 days culture period (Figure 2B,C). It was observed that SMCs were homogeneously distributed within the GelMA hydrogel on the initial day, along with a clear separation of HUVECs and SMCs about 100 µm for effective mass transportation. In addition, the fluorescence area and integrated optical density (IOD) of HUVECs showed obvious increasing trends, of which SMCs showed a slight increase for 5 days culture period (Figure 2D). The highly hydrophilic GelMA hydrogel offered great possibilities for HUVEC adhesion and proliferation on the surface, and the SMCs encapsulated in the GelMA hydrogel also showed good proliferation during culture, exhibiting a sign of the early maturation of the artificial arterial wall.^[^ ^16^ ^]^ The arterial intima‐media interface was maintained during the 5‐day culture period, which was necessary for retaining the physiological structure of the arterial wall as well as the native vasculature. The results show that GelMA hydrogel well‐confined HUVECs and SMCs in our engineered arterial model without affecting cell growth, and the spatial distribution of HUVECs and SMCs was achieved, mimicking the native structure of arterial walls.

Further confirmation of cell activities and cellular functions within the engineered arterial wall were examined by high‐resolution fluorescence images of cell actin and specific protein expressions of HUVECs and SMCs, respectively. Considering the properties of surface adhesion of HUVECs, HUVECs cultured on 2D plastic plates served as control. As shown in Figure 2E–G, the well‐defined green fluorescence of HUVEC‐actin indicated that the HUVECs exhibited an aligned morphology on the surface of GelMA hydrogel.^[^ ^12a^ ^]^ Vascular endothelial‐cadherin (VE‐Cad), expressed on the membranes of HUVECs, plays a crucial role in maintaining a restrictive endothelial barrier and vascular stability.^[^ ^17^ ^]^ It was evidenced that the VE‐Cad fluorescence of HUVECs (purple) also showed highly organized networks (Figure 2H–J), possibly due to the ECM remodeling by the presence of GelMA hydrogel. In addition, the visibly increased fluorescence levels in the purple of VE‐Cad indicated that the organization of HUVECs was augmented with increased culture time. However, the organization and networks of HUVECs were limited in the 2D culture, while the cells showed a good proliferation, which might be due to the insufficient ECM in the 2D plates.^[^ ^18^ ^]^ Further, confocal images of actin and alpha‐smooth muscle actin (*α*‐SMA) in SMCs, which was essential for their motility, structure, and integrity, indicated the formation of a 3D cell arrangement within the GelMA hydrogel (Figure 2K–M).^[^ ^7a^ ^]^ It should be noted that the overlay image processing technique used for observing all 3D cultured SMCs within the GelMA matrix might have obscured the visibility of certain 3D actin structures formed by the cells during imaging. Thus, the individual layer‐by‐layer images could be found in Figure S1, Supporting Information, to ensure a comprehensive analysis of the 3D actin structures. Together, GelMA hydrogel showed great potential in promoting cell growth and cytoskeletal organization, as well as creating excellent ECM surroundings.^[^ ^18^, ^19^ ^]^ The biometric arterial wall reproduced individual counterparts of the vasculature including endothelium, subendothelial ECM, and tunica media.^[^ ^7^, ^17^ ^]^

### 2.2 The Altered SMC Phenotype, Dysfunction of Endothelium, and Bioefficacy Assessments of the Engineered 3D AS Model

To stimulate AS pathological environments, hyperlipidemic surroundings and inflammatory stimulation were determined as key inducers for the development of pathological arterial walls.^[^ ^7^, ^12^ ^]^ It is well established that AS development mainly relies on atherogenic cholesterol‐containing lipoprotein cholesterol (LDL) that is subsequently oxidized in the form of ox‐LDL and easily taken up by cells to form atherosclerotic plaques or lesions in the intima. In addition, ox‐LDL effectively causes apoptosis at concentrations of 50 µg mL^−1^ and more without causing cytotoxicity as reported.^[^ ^7c^ ^]^ Moreover, inflammatory cytokines, such as TNF‐*α* (2 ng mL^−1^) and IL‐1*β* (2 ng mL^−1^), directly play a pro‐inflammatory role in inducing abnormal lipid and carbohydrate metabolism, thereby accelerating the development of AS.^[^ ^20^ ^]^ Notably, the cell viabilities of HUVECs and SMCs were assessed, respectively, and it was observed that the cells were maintained at above 80% in all treatments after 24 and 48 h incubation, suggesting no obvious cell cytotoxicity in the presence of ox‐LDL (50 µg mL^−1^), TNF‐*α* (2 ng mL^−1^), and IL‐1*β* (2 ng mL^−1^) (Figure S2, Supporting Information). Herein, ox‐LDL (50 µg mL^−1^), TNF‐*α* (2 ng mL^−1^), and IL‐1*β* (2 ng mL^−1^) were considered as the hyperlipidemic and inflammatory conditions as well as AS pathological environments.

**Figure 3:**
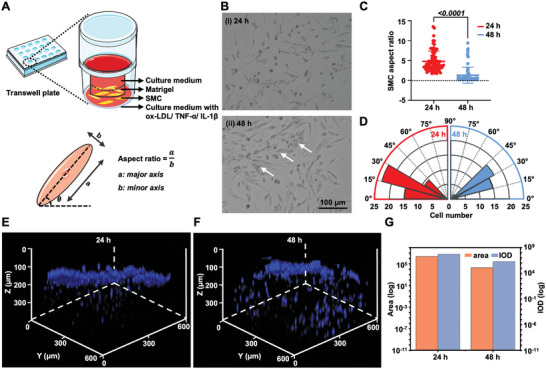
The altered SMC phenotype. A) Schematic illustrations for investigation and characterization of SMC immigration using Transwell plate. B) Brightfield images of migrated SMCs and C,D) quantification on the bottom chamber of the Transwell plate in terms of SMC aspect ratio and angle orientation. E,F) Overlay 3D‐reconstructed CLSM images for SMC‐loaded matrigel and G) quantification of fluorescence area and IOD of the nucleus.

We investigated the activities and functions of SMCs and HUVECs in the 3D arterial wall model under the treatment of ox‐LDL, TNF‐*α*, and IL‐1*β*. The phenotypic transformation of SMCs from the quiescent state to the migration state is a crucial cause of AS.^[^ ^21^ ^]^ Healthy SMCs are mainly contractile, and their main function is to maintain the elasticity of blood vessels. However, SMCs proliferate and migrate to the intima upon external stimuli, exhibiting a proliferative and migrated phenotype.^[^ ^22^ ^]^ We employed the Transwell approach to study the altered SMC phenotype after stimulation by hyperlipidemic and inflammatory conditions (**Figure** **3**A). The migrated SMCs on the bottom of the Transwell plate were captured using a microscope, and the aspect ratio and angle orientation of the cells were then quantified after 24 and 48 h incubation. SMCs tend to display a short spindle‐shaped morphology after 24 h incubation while exhibiting a less polarized and rhomboidal cell shape (white arrows) after 48 h incubation (Figure 3B). Besides the significant decrease in SMC aspect ratio (*P* < 0.0001) with the prolonged treatment (Figure 3C), the SMC angle orientation was gradually transformed, demonstrating the alternate SMC phenotype from the relatively quiescent state toward the migrated state (Figure 3D).^[^ ^12b^ ^]^ To further validate the migration of SMCs induced by hyperlipidemia and inflammatory factors, cell nuclei of SMCs were analyzed in 3D‐reconstructed CLSM images for 4′,6‐diamidino‐2‐phenylindole (DAPI)‐stained matrigel in Transwell plates. As shown in Figure 3E–G, the blue fluorescence of DAPI obviously shifted downward within the matrigel, along with the increased fluorescence area and IOD with prolonged culture time, reflecting a deeper SMC migration due to the stimulation of hyperlipidemia and inflammatory factors. These results indicated that the SMCs were activated and altered from the quiescent state to the relative migration state.^[^ ^7a^ ^]^

**Figure 4:**
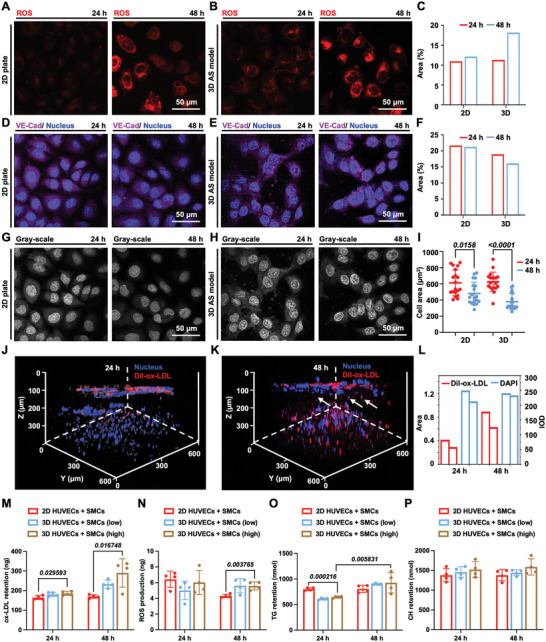
Dysfunction of the endothelium, and bioefficacy assessments of the 3D AS model. A,B) The ROS detection of HUVECs in 2D plate culture and 3D AS model and C) quantification of fluorescence area. Hyperlipidemic conditions induced an increase in ROS levels of HUVECs both in the 2D plate culture and 3D AS model. D,E) CLSM images showing VE‐cad under pathological treatment both in the 2D plate culture and 3D AS model and F) quantification of fluorescence area. G,H) The gray‐scale images of VE‐cad expression and I) measurement of cell area at different time points. J–L) Overlay CLSM images of nucleus and Dil‐ox‐LDL after 24 and 48 h incubation, respectively, and M–P) the level of ox‐LDL, ROS, TG, and CH measured by ELISA using the 3D cultured cells incubated with a high concentration of hyperlipidemic and inflammatory factors (ox‐LDL, 50 µg mL^−1^; TNF‐*α* at 2 ng mL^−1^, and IL‐1*β* at 2 ng mL^−1^), along with the 3D cultured cells incubated with a low concentration of hyperlipidemic and inflammatory factors (ox‐LDL, 25 µg mL^−1^; TNF‐*α* at 1 ng mL^−1^, and IL‐1*β* at 1 ng mL^−1^) and 2D plate culture as controls.

Inflammatory cytokines, such as TNF‐*α* and IL‐1*β*, induce increased EC oxidative stress, promoting inflammatory response and endothelial dysfunction.^[^ ^23^ ^]^ In turn, apoptosis and EC damage caused by inflammatory factors further aggravate ox‐LDL accumulation, promoting lipid deposition and the progression of AS.^[^ ^24^ ^]^ Accordingly, in our experimental model, the level of reactive oxygen species (ROS) produced by HUVECs within the engineered arterial wall was investigated after incubation with TNF‐*α* (2 ng mL^−1^), IL‐1*β* (2 ng mL^−1^), and ox‐LDL (50 µg mL^−1^) at the determined time points, along with the 2D plate culture as the control groups (**Figure** **4**A–C). A very weak production of ROS was detected after 24 h incubation, while it was obviously increased both in 2D and 3D culture groups after stimulation for 48 h. In addition, a decreased level of VE‐cad expression, the endothelia‐specific functional protein of HUVECs, was observed both in 2D and 3D culture groups, demonstrating the loss of endothelial functions to some extent (Figure 4D–F).^[^ ^12b^ ^]^

We further investigated the cell morphology by cell area using the gray‐scale images with clear cell boundaries to study the damaging effect of hyperlipidemic and inflammatory factors on HUVECs (Figure 4G–I).^[^ ^7c^ ^]^ The average cell area of HUVECs in 2D plate culture significantly decreased after 48 h incubation (*P* = 0.0158). The average cell area of HUVECs in the 3D model decreased more significantly than that of cell area in the 2D culture, indicating a higher sensitivity and more effective infiltration of the 3D model for the atherogenic agents than the 2D cell culture.^[^ ^25^ ^]^ Moreover, the ox‐LDL labeled with 1, 1'‐dioctadecyl‐3, 3, 3', 3'‐tetramethyl indocarbocyanine perchlorate (Dil‐ox‐LDL) was tracked in the 3D model after 24 and 48 h of incubation. It was found that both the fluorescence intensity and area of Dil‐ox‐LDL showed an increasing trend with the extended incubation period of 48 h, demonstrating the retention of ox‐LDL within GelMA hydrogel (Figure 4J–L). Interestingly, in line with preceding SMC migration results, the Dil‐ox‐LDL (red fluorescence) and nucleus (blue fluorescence) appeared in the blank GelMA layer (model intima‐media‐interface) after 48 h of incubation (white arrows), which implied the migration of SMCs.^[^ ^12b^ ^]^ Consequently, these results, the increased level of ROS, decreased expression of VE‐cad, and retention of ox‐LDL, indicated that the inflammatory surroundings and dysfunction on HUVECs were successfully recapitulated using our model.^[^ ^6^ ^]^

To further validate the bioefficacy of the engineered 3D AS model, the level of ox‐LDL, ROS, human triacylglycerol (TG), and human cholesterol (CH) were measured and compared with a coculture of HUVECs and SMCs on the 2D plate as control groups, emphasizing the significance of incorporating vascular architectures and pathological features in the biometric AS disease models toward the evaluation of zinc ions. Notably, enzyme‐linked immunosorbent assays (ELISA) provide a highly sensitive and specific detection for analyzing the level of protein, allowing for accurate quantification of target proteins in the samples. As shown in Figure 4M,N, the ox‐LDL retention and ROS production were higher in the 3D AS model after 48 h of incubation compared with the group incubated with a low concentration of ox‐LDL, TNF‐*α*, and IL‐1*β* and the 2D cultured cells. In addition, the increased ox‐LDL retention and ROS production in the group of 3D cultured cells attributed to the promoted infiltration and diffusion of cell cytokines and differentiation of the cells within GelMA hydrogel. Specifically, TG, a risk factor for CVD, was illustrated as significantly increased after 48 h of pathological stimulation (*P* = 0.0058) in the constructed 3D AS model, indicating the promoted AS progression (Figure 4O).^[^ ^26^ ^]^ The increased production of TG might also chelate divalent Zn^2+^, leading to the lower bio‐availability of the zinc ions in the 3D AS model.^[^ ^27^ ^]^ In addition, a slight increase in CH was observed both in the 3D AS model and 2D culture (Figure 4P) along with the group incubated with a low concentration of ox‐LDL, TNF‐*α*, and IL‐1*β* and the 2D cultured cells, demonstrating the excess lipid accumulation and disorder of metabolism of incubated cells.^[^ ^28^ ^]^ Taken together, cell morphology and biological functions of the engineered 3D arterial wall successfully exhibited key pathological features with the combination of hyperlipidemic and inflammatory stimulations. The pathological cell morphology was shown including the evident HUVECs damage analyzed by the expression of VE‐Cad, promoted inflammatory response detected by the secretion of ROS, and altered SMCs phenotype observed by the immigration of SMCs. Biological functions of the diseased 3D arterial wall exhibited key pathological features evidenced by the higher expression of ROS, ox‐LDL, CH, and TG with the combination of hyperlipidemic and inflammatory stimulations. Overall, we believe that ox‐LDL (50 µg mL^−1^), TNF‐*α* (2 ng mL^−1^), and IL‐1*β* (2 ng mL^−1^) could provide the desired pathological microenvironments in the engineered 3D AS model in vitro.

### 2.3 Evaluation of Zinc Ions at Various Concentrations

**Figure 5:**
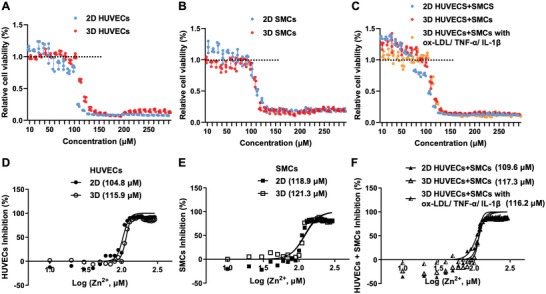
Zinc ion sensitivity of 3D‐cultured individual cells, normal arterial wall model, and constructed pathological arterial wall model, along with the 2D‐cultured cells as the comparison. A–C) Relative cell viability after incubation with zinc ions at different concentrations ranging from 0 to 300 µm. D–F) Dose‐response of 2D‐ and 3D‐cultured cells to zinc ions based on the cytotoxicity tests. The IC_50_ values for 2D‐ and 3D‐cultured HUVECs were 104.8 and 115.9 µm, respectively, and that of SMCs were 118.9 and 121.3 µm, respectively. The IC_50_ values for the coculture of SMCs and HUVECs under 2D culture, normal 3D arterial wall, and 3D AS model were 109.6, 117.3, and 116.2 µm, respectively.

During the preclinical evaluation of implanted scaffolds and vascular stents, the cytotoxicity approaches are primarily executed using vascular cells to predict the effect of degradation products on cells.^[^ ^2^ ^]^ Zinc ions, the main degradation products of zinc‐based absorbable biomaterials, were considered an important factor in the regulation of SMC and EC growth and functions.^[^ ^4b^ ^]^ Initially, to ensure the effects of zinc ions, the cell number of SMCs and HUVECs was assessed by exposing them to the culture medium with zinc ions ranging from 0 to 300 µm using the cell counting kit (CCK)‐8 assay in 2D culture and 3D culture using GelMA (**Figure** **5**A,B). The cell number in all groups showed a concentration dependency on the zinc ions. Zn^2+^ ion concentrations below 50 µm increased the HUVEC viability above 100% compared to the baseline, implying an increased cell number after 24 h incubation. The same trend was observed for the individual culture of SMCs while the concentration of zinc ions was lower than 70 µm, which was quite close to the results as reported.^[^ ^7b^ ^]^

Then, the Zn^2+^‐sensitivities of cocultures of SMCs and HUVECs in the 2D plate, engineered 3D arterial wall, and 3D AS model were analyzed. As depicted in Figure 5C, after incubation with zinc ions, enhanced cell numbers were observed both in 2D culture (below 70 µm) and engineered 3D arterial wall (below 80 µm). The slightly distinct concentration ranges might be due to the GelMA, which may affect the diffusion of zinc ions.^[^ ^9^ ^]^ Notably, the proliferation profile remained above the baseline (100%) for zinc ion concentrations up to 100 µm, indicating an enhanced cell number within the 3D AS model as well as exhibiting a great potential of zinc ions for vascular regeneration and angiogenesis in vivo within pathological environments.^[^ ^4c^ ^]^ The highest cell numbers were reached for Zn^2+^ concentrations around 20.0 µm within the 3D AS model, which was determined as one typical low concentration (20.0 µm) for maximum cell‐number‐promoting effect for further investigations.

Further, the best‐fit half‐maximal inhibitory concentration (IC_50_) values of zinc ions were analyzed in all groups based on the CCK‐8 cytotoxicity results (Figure 5D–F). The IC_50_ values for 2D‐ and 3D‐cultured HUVECs were 104.8 and 115.9 µm, respectively, while that of SMCs were 118.9 and 121.3 µm, respectively. The IC_50_ values for the coculture of SMCs and HUVECs under 2D culture and engineered 3D arterial wall were 109.6 and 117.3 µm, respectively. The employment of GelMA hydrogel might have led to the difference in the eventual availability of zinc ions compared to the 2D monolayers.^[^ ^9^, ^15^ ^]^ The IC_50_ value of zinc ions in the 3D AS model was at the concentration of 116.2 µm, slightly lower than the healthy engineered arterial wall (117.3 µm). The cells within the pathological environments might be more sensitive and vulnerable, further leading to a slight decrease in the IC_50_ value.^[^ ^29^ ^]^ Together, the higher IC_50_ values analyzed using our engineered 3D tissue models implied potential significance for better‐differentiated states of the cells and promoting the accuracy of the zinc ion evaluation based on 2D cell monolayers.

Considering the biphasic effect of zinc ions on cell proliferation, cell cycle and apoptosis investigations were conducted at one typical low concentration (20.0 µm) with the maximum cell‐promoting effect and one typical high concentration (116.2 µm) for best‐fit IC_50_ value in the 3D AS model. Investigating the cell cycle is critical in comprehending the complex mechanisms that regulate cell growth and identifying specific drugs and therapeutic targets that are evolved in cell cycle for the treatment of diseases, which are essential for maintaining healthy tissues.^[^ ^30^ ^]^ Prior to the zinc ion treatment in the 3D AS model, the cell cycle and apoptosis of HUVECs and SMCs under hyperlipidemic and inflammatory conditions were investigated separately (Figures S3 and S4, Supporting Information). In the ox‐LDL/TNF‐*α*/IL‐1*β*‐treated groups, the percentages of HUVECs in the G0G1‐phase were increased from 48.16% to 57.74% and 38.98% to 44.42% in 2D‐ and 3D‐cultured HUVECs, respectively, implying inhibited cell transition from the G0G1‐phase to the S‐phase and G2M‐phase during the DNA synthesis process to some extent, which was consistent to previously reported data (Figure S3, Supporting Information).^[^ ^31^ ^]^ Contrarily, the percentage of SMCs in the G0G1‐phase declined from 65.15% to 51.68% and 51.32% to 41.60% in 2D‐ and 3D‐cultured SMCs, respectively, along with a distinct increase from 30.29% to 44.84% and from 43.97% to 48.76% of SMCs in the S‐phase in 2D‐ and 3D‐cultured HUVECs, respectively, which is attributed to the phenotypic switch of SMCs toward the proliferative state.^[^ ^7b^ ^]^ In apoptosis investigations, the cells within Q1, Q2, Q3, and Q4 zones indicate the dead cells or cell fragments, late‐apoptotic, early‐apoptotic, and live cells, sequentially. The treatment of ox‐LDL/TNF‐*α*/IL‐1*β* induced an increase in the early apoptosis (Q3) of HUVECs from 9.73% to 15.9% and 5.73% to 8.35% in 2D‐ and 3D‐cultured HUVECs, respectively, demonstrating some degree of endothelial dysfunctions (Figure S4, Supporting Information).^[^ ^4^, ^9^ ^]^ Moreover, zinc ions induced markable cell death at the concentration of 116.2 µm, while the cell percentage (Q2) increased from 3.10% to 35.3% and 2.86% to 32.4% in 2D‐ and 3D‐cultured HUVECs, respectively. The same trend was also observed in SMC groups, while the cell percentage (Q2) increased from 1.99% to 15.8% and 1.10% to 20.6% in 2D‐ and 3D‐culture, respectively.

**Figure 6:**
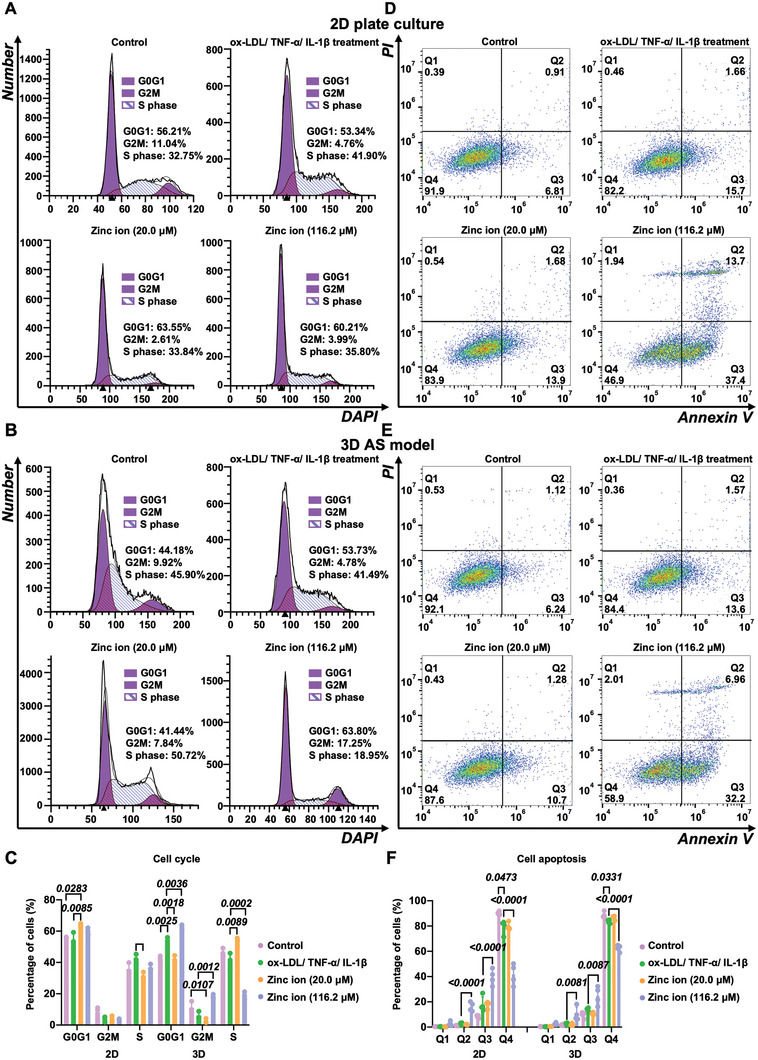
Cell cycle and apoptosis evaluations. A–C) Cell cycle variations and D–F) cell apoptosis observations for zinc ions at one typical low concentration (20.0 µm) for maximum cell‐number‐promoting effect and one typical high concentration (116.2 µm) for best‐fit IC_50_ value using 3D AS model (*n* = 3). Cells within Q1, Q2, Q3, and Q4 zones indicating the dead cells or cell fragments, late‐apoptotic, early‐apoptotic, and live cells, sequentially.

Subsequently, we investigated the cell cycle in the engineered 3D AS model, along with the 2D coculture of SMCs and HUVECs as the control group. Based on the individual cell cycle analysis, the dysfunction of HUVECs leads to an increase in the G0G1‐phase of HUVECs, while the SMCs with proliferative phenotype showed a downward trend in the G0G1‐phase. So far, only a few studies have investigated the cell cycle for the coculture of HUVECs and SMCs, although it appears crucial for exploring cell–cell interactions during AS progression. As shown in **Figure** **6**A, a slight decrease in the G0G1‐phase was observed from 56.21% to 53.34% in the coculture of HUVECs and SMCs on the 2D plate. However, a significant increase from 44.18% to 53.73% (*P* = 0.0025) in the G0G1‐phase of the 3D AS model was detected (Figure 6B,C). In the layered arterial wall, hyperlipidemic and inflammatory factors were initially encountered and heavily phagocytosed by HUVECs on the surface of GelMA before penetrating to SMCs in the tunica media. Accordingly, the chronological phagocytosis process of cell cytokines within the constructed 3D AS model led to a predominant response of the HUVECs in the model. Moreover, a significant amount of HUVECs entered from the G0G1‐phase (*P* = 0.0018) to the S‐phase (*P* = 0.0089) incubated with zinc ions at the concentration of 20 µm using the 3D AS model. However, the amount of HUVECs in the S‐phase decreased significantly upon incubation with zinc ions at the concentration of 116.2 µm (*P* = 0.0002), along with a significant increase in the G0G1 phase (*P* = 0.0036), demonstrating that the zinc ions inhibited DNA synthesis in hyperlipidemic and inflammatory factor‐treated 3D AS model via inhibition of cell transition from the G0G1 to the S phase of the cell cycle.

We further examined the apoptosis of the 3D AS model incubated with the zinc ions at various concentrations (20.0 and 116.2 µm), along with the 2D culture as the control groups (Figure 6D–F). The 3D AS model with zinc supplementation (20.0 µm) displayed a slight increase from 84.4% to 87.6% in Q4 (live cells). The toxicity of zinc ions at the concentration of 116.2 µm was confirmed by the significant increase in the percentage of cells in Q2 (dead cells) both in 2D cocultured cells (*P* < 0.0001) and 3D AS model (*P* = 0.0081), the significant increase in the percentage of cells in Q3 (early apoptosis cells) in 2D cocultured cells (*P* < 0.0001) and 3D AS model (*P* = 0.0087), as well as the significant decrease of the percentage of cells in Q4 (live cells) in the 2D cocultured cells (*P* < 0.0001) and the 3D AS model (*P* < 0.0001). These results suggested that the 3D AS model highly mimics the interactions between tissues and cells, thus providing an excellent platform for evaluating cytokines.

### 2.4 The Immune Response of Macrophages after Treatment with Zinc Ions

Macrophages (MCs) play a critical role in the innate immune system and in maintaining tissue homeostasis.^[^ ^17^ ^]^ In addition to diverse tissue‐specific roles, MCs are highly sensitive and can alter their phenotype in response to environmental signals.^[^ ^32^ ^]^ Specifically, MC subtypes can be generally grouped into either of two classes including the M1 pro‐inflammatory role and M2 anti‐inflammatory role.^[^ ^17^ ^]^ In the early stage of AS, M1 MCs are involved in the phagocytosis of ox‐LDL, which contributes to the production of pro‐inflammatory cytokines and the formation of foam cells.^[^ ^33^ ^]^ As atherosclerotic lesions progress, the MCs can shift to M2 phenotype along with the microenvironment changes, leading to the upregulation of scavenger receptors that are involved in the clearance of apoptotic cells.^[^ ^33^ ^]^ Targeting the protein markers associated with MC phenotypes alternates, such as cluster differentiation of 86 (CD86) and interleukin (IL)‐6 for M1 polarization and human mannose receptor (CD206) and IL‐10 for M2 polarization, is broadly used for identifying MC subtypes.^[^ ^17^ ^]^

**Figure 7:**
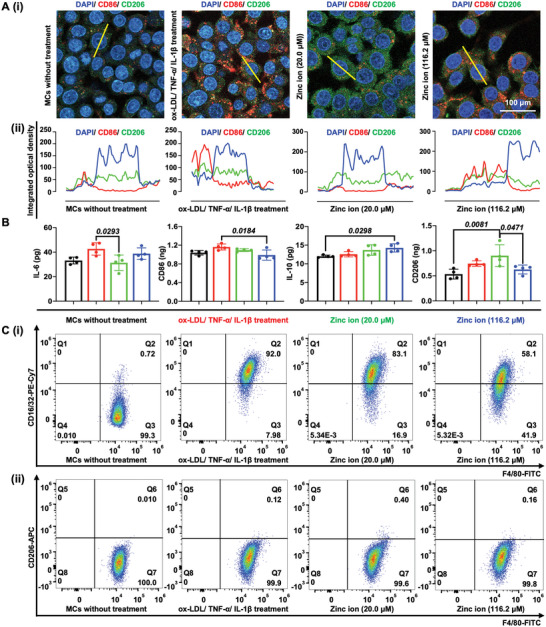
The effect of hyperlipidemic and inflammatory factors following treatment by zinc ions at various concentrations (20.0 and 116.2 µm) on the M1 and M2 polarization of MCs. A) (i) CLSM images showing the expression of CD86 and CD206 and (ii) quantification of cross‐sectional fluorescence intensity profile based on the yellow line in (i). B) Investigation of macrophage markers, including genetic markers associated with cellular pillarization including IL‐6 and CD86 for M1 markers, and IL‐10 and CD206 for M2 using ELISA. C) Flow cytometry analysis of MCs infiltration (F4/80^+^), showing (i) M1‐like macrophages (F4/80^+^ and CD16/32^+^) and (ii) M2‐like macrophages (F4/80^+^ and CD206^+^).

To explore the MC polarization after incubation with ox‐LDL/TNF‐*α*/IL‐1*β*, following treatment with the zinc ions at 20.0 and 116.2 µm, respectively, we employed immunofluorescence, ELISA, and flow cytometry to categorize the MC subtypes. Initially, it was observed that the expression of CD86 (red) increased after incubation with ox‐LDL/TNF‐*α*/IL‐1*β*, followed by a decrease upon treatment of zinc ions at the concentration of 20.0 µm (**Figure** **7**A). In addition, the expression of IL‐6 increased after incubation with ox‐LDL/TNF‐*α*/IL‐1*β* and decreased significantly (*P* = 0.0293) after the treatment with 20.0 µm zinc ions, along with the increased expression of IL‐10, indicating M2 polarization (Figure 7B). Further, we verified ox‐LDL/TNF‐*α*/IL‐1*β*‐induced M1 polarization of MCs based on the expression of CD86 by flow cytometry analysis, following a decrease after treatment with 20.0 µm zinc ions (Figure 7C). The release of the inflammatory cytokines and expression of surface markers confirmed the polarization of MCs into pro‐inflammatory M1 cells after ox‐LDL/TNF‐*α*/IL‐1*β* treatment, and their subsequent downregulation demonstrated the regulatory role of 20.0 µm zinc ions. This process further confirmed the development of pathological features of AS and the inflammatory inhibition effect of zinc ions at the concentration of 20.0 µm to some extent. Taken together, zinc homeostasis in MCs is promising for immunity.

### 2.5 Gene Ontology Function Analysis

**Figure 8:**
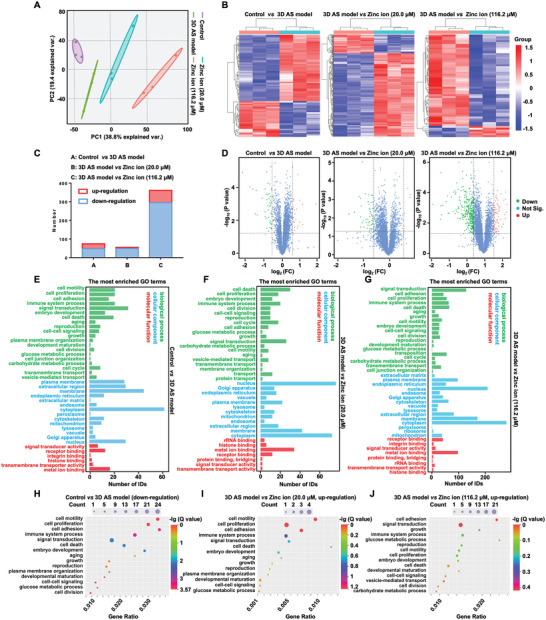
Pathway‐level comparisons between datasets and GO analysis. The control groups included the comparison between the normal arterial wall and the 3D AS model, 3D AS model and 3D AS model with zinc ion treatment at the concentration of 20 µm, and 3D AS model and 3D AS model with zinc ion treatment at the concentration of 116.2 µm. A) PCA analysis indicating all merged proteomic in individual datasets. B) Heat maps illustrating the differences in expression of proteins across various control groups. C) Statistical analysis of the number of differential proteins in each group. D) Volcano plots indicating significantly altered proteins in the comparison of individual control groups. Negative log‐transformed *P*‐values (two‐tailed Student's *t*‐test) associated with individual proteins (circle) against the difference in means of log2‐transformed normalized values for individual proteins. E–G) The most enriched GO terms, indicating differences in biological processes, cellular components, and molecular function in distinct control groups. H–J) Significantly altered gene ratio associated with the biological process in various control groups (*P* < 0.05).

Prior to the GO and KEGG comparison, we made the comparison between the normal arterial wall and the 3D AS model, 3D AS model and 3D AS model with zinc ion treatment at the concentration of 20 µm, and 3D AS model and 3D AS model with zinc ion treatment at the concentration of 116.2 µm based on the statistical analyses of the merged proteomic were obtained using the tandem mass tags (TMT) strategy and visualized using principal component analyses (PCA) and hierarchical clustering heat maps. PCA indicated all merged proteomics in individual datasets with clear class separation and accounted for 38.8% of the variance in gene expression, demonstrating the profound changes and independent gene expression in each group due to the stimulation of pathological factors and zinc ion treatments (**Figure** **8**A).^[^ ^34^ ^]^ In addition, individual groups exhibited consistent and distinct changes when comparing samples in terms of both global protein expressions (Figure 8B). Overall, a consistent decrease in the global protein was observed (Figure 8C,D). The differential expressed proteins in the 3D AS model with or without zinc ion treatment was linked to a great amount of GO terms according to the GO function analysis, including biological function (green), cellular component (blue), and molecular function (red) (Figure 8E–G). In addition, the number of proteins enriched in biological functions is the largest (green). Accordingly, we further detected the significant downregulation and upregulation of biological process terms in each group. It was demonstrated that the cell motility, cell proliferation, and cell adhesion showed downregulation trends while the healthy arterial incubated with inflammatory and hyperlipidemic cytokines, indicating the formation of the pathological environments as well as the endothelium dysfunction within the engineered 3D AS model (Figure 8H). The downregulated cell proliferation and cell adhesion cause apoptosis, as demonstrated in Section 2.3.^[^ ^34^ ^]^ However, cell motility, cell proliferation, and cell adhesion exhibited significantly upregulated after zinc ion treatment at the concentration of 20.0 µm (*P* < 0.05), which testified to the CCK‐8 results as well as the growth‐promoting effect on cells (Figure 8I). Moreover, the signal transduction was significantly upregulated in the group of zinc ion treatment at the concentration of 116.2 µm compared with the engineered 3D AS model, implying the potential abnormal signaling and thereby reduced cell activity (Figure 8J).

### 2.6 KEGG Pathway Analysis and Comparison

**Figure 9:**
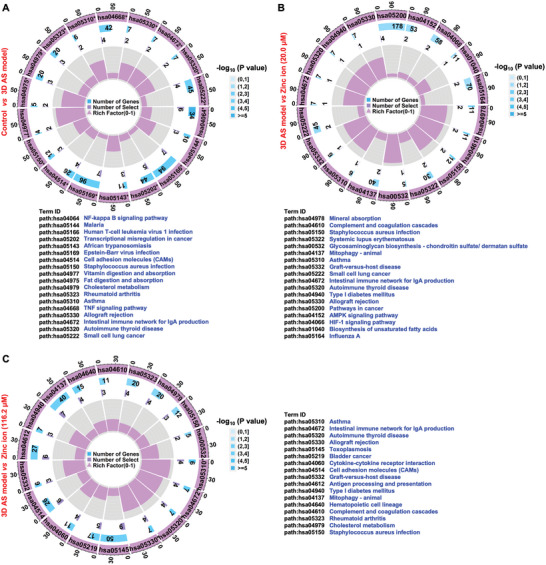
KEGG pathway analysis and comparison. The most concerned KEGG pathways and related gene signals associated with the identified proteins with the comparison between A) the normal arterial wall and the 3D AS model, B) the 3D AS model and 3D AS model with zinc ion treatment at the concentration of 20 µm, and C) 3D AS model and 3D AS model with zinc ion treatment at the concentration of 116.2 µm.

KEGG pathway analysis of the 3D AS model treated with inflammatory and hyperlipidemic cytokines revealed significantly altered 41 pathways, of which the top five closely related pathways included NF‐kappa B signaling pathway, malaria, human T‐cell leukemia virus 1 infection, transcriptional misregulation in cancer, and African trypanosomiasis (**Figure** **9**A). Among others, NF‐kappa B is a core regulatory signaling pathway member in terms of apoptosis, and the downregulated NF‐kappa B potentially implied the disturbance of homeostasis.^[^ ^35^ ^]^ In addition, the intercellular cell adhesion molecule‐1 (ICAM1), one of the gene symbols closely associated with NF‐kappa B, was known as a crucial adhesion molecule that mediated cell‐to‐cell or cell‐to‐matrix contact and binding, thereby participating in pathological processes such as cell signaling and activation, cellular tissue growth and differentiation, immune response, inflammatory response, and angiogenesis.^[^ ^36^ ^]^ ICAM1 was strongly expressed on normal HUVECs, as reported, and the downregulation of ICAM1 might indicate the dysfunction of the endothelium within the engineered 3D AS model.^[^ ^37^ ^]^ Moreover, ICAM1 was likewise related to the other top five signaling pathways, malaria, human T‐cell leukemia virus 1 infection, and African trypanosomiasis, suggesting its meaningful role in biological activities. These results showed the role of ICAM1 as a potential target in the development of AS.

To further analyze the biphasic effect of zinc ions, the KEGG pathway variation was investigated in the 3D AS model with 20.0 and 116.2 µm zinc ion treatment, respectively. As shown in Figure 9B, a significantly decreased expression of mineral absorption was observed that was associated with solute carrier family 30 member 1 (SLC30A1) and heme oxygenase‐1 (HMOX1) gene symbols (*P* < 0.05). Notably, SLC30A1 is expressed at the cell membrane and encodes a putative zinc transporter.^[^ ^34^ ^]^ Besides that, significant downregulation of HMOX1 was involved (*P* < 0.05). Some researchers found that the HMOX1 inhibitor could significantly alleviate the occurrence and development of ferroptosis in mouse hearts and thus protect cardiac function. However, the HMOX1 upregulation mediated the release of free iron ions from heme, which accumulate in cardiomyocytes and induce ferroptosis.^[^ ^38^ ^]^ Although the precise biological function of HMOX1 in the AS progression is poorly understood, our results indicated that HMOX1‐downregulated expression was potentially essential in reversing AS progression.^[^ ^39^ ^]^ Further, KEGG comparisons with zinc ions treatment at the concentration of 116.2 µm revealed a mark of significantly altered asthma, the intestinal immune network for IgA production, autoimmune thyroid disease, allograft rejection, and toxoplasmosis pathways (*P* < 0.05), which was regulated by almost coherent gene symbols (Figure 9C). These included decreased human leukocyte antigen‐G (HLA‐G), protection from immune cell attack of maternal origin,^[^ ^40^ ^]^ human leukocyte antigen‐C (HLA‐C), inducing allogeneic immune cell responses,^[^ ^41^ ^]^ human leucocyte antigen‐E (HLA‐E), regulating biological activities of the natural killer cell,^[^ ^42^ ^]^ and increased clusters of differentiation 40 (CD40), mediating a broad immune‐inflammatory response.^[^ ^43^ ^]^ Together, our 3D AS model provides an extremely promising new strategy and an excellent platform for the evaluation of zinc ions and other metal ions as well as the discovery of new targets for the clinical therapy of AS.

## 3 Conclusions

In summary, we developed a 3D arterial wall model with incorporated HUVECs and SMCs within a defined multi‐layer space using GelMA hydrogel, thus providing a biomimetic platform to comprehend AS progression and the evaluation of zinc ions. Initially, the cross‐linking and stacking of GelMA hydrogels produced a biocompatible environment and multi‐layer fixation of various cell types. HUVECs were seeded on the surface of GelMA to generate vascular tubes as well as the vascular barrier. The presence of blank GelMA mimicking arterial intima‐media supplied space for SMCs immigration. Additionally, the treatment of ox‐LDL, TNF‐*α*, and IL‐1*β* stimulated the engineered arterial wall, exhibiting necessary pathological features, such as significantly higher expression of ROS, ox‐LDL, CH, and TG, along with the 2D‐cultured cells as the comparisons, indicating the formation of the 3D AS model. Subsequently, the engineered 3D AS model was employed to analyze the biphasic effect of zinc ions. The maximum cell‐promoting effect was achieved with zinc ion incubation at the concentration of 20.0 µm. Notably, higher IC_50_ values of the 3D AS model (116.2 µm) implied potential significance for promoting the accuracy of the zinc ion evaluation compared with the 2D cell monolayers (109.6 µm). Cell cycle results demonstrated that zinc ion inhibited DNA synthesis in hyperlipidemic and inflammatory factor‐treated 3D AS models via inhibition of cell transition from the G0G1 to the S phase of the cell cycle.

Furthermore, GO analysis showed that the effect of zinc ions was highly related to biological processes of cell motility, cell proliferation, and cell adhesion. More importantly, the variation of the KEGG pathway implied potential targets, such as SLC30A1 and HMOX1 at the low concentration (20.0 µm) with maximum cell‐promoting effect, and HLA‐G, CD40, HLA‐C, and HLA‐E at the high concentration (116.2 µm) corresponding to the IC_50_ value in the 3D AS model. However, in‐depth verification of various gene symbols is still required to investigate the AS progression and molecular mechanisms of zinc ions. Together, we provide an attractive evaluation platform based on the diseased 3D arterial wall that was expected to serve as an advanced tool for studying CVD progression, evaluation of zinc ions as well as eluted drugs from vascular stents, thus promoting the application of zinc‐based absorbable biomaterials toward the clinical therapy in AS.

## 4 Experimental Section

### Materials

GelMA with photoinitiator, lithium phenyl‐2,4,6‐trimethylbenzoylphosphinate, was purchased from Intelligent Manufacturing Research Institute (IMRI, GM‐90, Suzhou, China). TNF‐*α*, ox‐LDL, and IL‐1*β* were purchased from Sigma‐Aldrich (St. Louis, USA). DAPI, HUVECs, human SMCs, and actin‐tracker green were purchased from Keygen Biotech Co., Ltd. (Nanjing, China). Paraformaldehyde (4% w/v), Triton X‐100, CCK‐8, and phosphate‐buffered saline (PBS) were purchased from Solarbio (Beijing, China). Mouse MCs were purchased from Kemao Biotechnology (Dongguan, China). Penicillin–streptomycin, Dulbecco's modified Eagle medium (DMEM), fetal bovine serum (FBS), and endothelial basal medium (F‐12K) were purchased from Gibco (Grand Island, USA). Cell labeling kits (Qtracker 525 and 655), *α*‐SMA, VE‐Cad primary polyclonal antibody, and goat anti‐rabbit IgG (H+L) secondary antibody were obtained from Thermo Fisher (Waltham, USA).

### Construction of the Artificial Arterial Wall

The culture medium for HUVECs and SMCs was F‐12K and DMEM, respectively, supplemented with 1% w/v penicillin–streptomycin and 10% w/v FBS, and incubated at 37 °C in 5% CO2 and 95% relative humidity. Basically, the artificial arterial wall comprised endothelium, tunica intima, and tunica media, formed by a HUVECs layer, pure GelMA hydrogel, and GelMA hydrogel with SMCs from inner to outer, respectively. Initially, SMCs were dispersed homogenously in GelMA polymer solution (10% w/v) at a final concentration of 4 × 10^6^ cells mL^−1^. The GelMA encapsulating SMCs was directly extruded within the inoculation round chamber and then cross‐linked using UV light (405 nm, 3 W) for 15 s. Notably, the surface tension of the hydrogel solution ensured that the extruded GelMA retained the cylindrical shape to allow photopolymerization. Subsequently, overlay and gelation of pure GelMA (5% w/v) occurred by UV light irradiation for 15 s. Subsequently, HUVECs were grown on the top surface of pure GelMA as the endothelium. Finally, the engineered arterial wall was incubated in the culture medium by mixing F‐12K and DMEM complete medium at a ratio of 1:1 until the SMCs fully aligned and HUVECs reached confluency.

To observe the cell distributions, the HUVECs and SMCs were labeled by the Qtracker 525 cell labeling kit and a Qtracker 655 cell labeling kit, respectively, based on the manufacturer's instructions for CLSM observations. To analyze the biological performances of cells, VE‐Cad expression, the cytoskeleton of HUVECs, *α*‐SMA expression, and the cytoskeleton of SMCs were investigated, respectively. Briefly, the samples were washed twice with PBS and fixed with paraformaldehyde (4% w/v) for 1 h. Then, the samples were soaked in 0.1% w/v Triton X‐100 (Aladdin) and 5% w/v bovine serum albumin (in PBS, Keygen) for 1 h at room temperature. The samples were added with VE‐Cad primary antibody (1:200 dilutions in PBS), or *α*‐SMA primary antibody (1:200 dilutions in PBS), and incubated overnight at 4 °C, respectively. Further, the goat anti‐rabbit IgG (H+L) secondary antibody (1:300 in PBS) was added for 2 h, and DAPI was added for 10 min at room temperature. Finally, the images were captured using CLSM after washing with PBS several times. For cytoskeleton staining, samples were fixed with paraformaldehyde (4% w/v) for 1 h, and cells were stained with the actin‐tracker, along with DAPI for nuclei. In brief, the sample was successively soaked in 0.1% w/v Triton X‐100 (Aladdin) for 10 min, actin‐tracker working solution for 20 min, and DAPI for 10 min at room temperature. The sample was scanned layer‐by‐layer for 3D construction and the obtained multiple images were overlayed using built‐in microscopy software. The fluorescence intensity profile along the line was measured using the built‐in software in the microscope (LSM900, Image system). The fluorescence area and IOD were measured by ImageJ software (Bethesda, USA).

### Recapitulation of Atherogenic Conditions and Biological Performances of Cells within the Pathological Arterial Wall

The experimental design simulated two key risks of AS, hyperlipidemic environment and inflammatory factors, as AS‐prone biochemical conditions. As for hyperlipidemia treatment, ox‐LDL was added to the culture medium at the concentration of 50 µg mL^−1^. To induce inflammation, TNF‐*α* and IL‐1*β* at the concentration of 2 ng mL^−1^, respectively, were added as proinflammatory agents. Initially, the HUVEC and SMC viabilities were investigated in the presence of ox‐LDL, TNF‐*α*, and IL‐1*β*, separately. In brief, 5000 cells per well were seeded into 96‐well microplates following incubation overnight. The cells were then subjected to treatment and incubation using a mixture of ox‐LDL (50 µg mL^−1^), TNF‐*α* (2 ng mL^−1^), and IL‐1*β* (2 ng mL^−1^) for a period of 24 and 48 h. After washing with PBS twice, CCK‐8 working solution (10 µL) was added to each well and incubated with fresh culture medium (100 µL) for 4 h. The optical densities at 450 nm of the supernatants were measured using a microplate reader (Thermo Fisher) with at least six parallel groups. By ensuring cell viability, the mixture of ox‐LDL (50 µg mL^−1^), TNF‐*α* (2 ng mL^−1^), and IL‐1*β* (2 ng mL^−1^) was employed to incubate the engineered arterial wall for another 2 days. For the 3D‐cultured groups, the cells were retrieved using GelMA lysate for subsequent analysis.

To explore the phenotype of SMCs altered by angiogenic agents, SMCs (5 × 10^4^ cells, 100 µL) were seeded in 24‐well Transwell polycarbonate membrane inserts (8 µm pore, Corning Incorporated, Corning, USA) which were coated with growth factor reduced matrigel (200 µg mL^−1^, 100 µL, Corning) at 37 °C. Fresh culture medium and culture medium with ox‐LDL (50 µg mL^−1^), TNF‐*α* (2 ng mL^−1^), and IL‐1*β* (2 ng mL^−1^) were added to the upper and bottom chambers, respectively, and then incubated for 24 and 48 h. The inserted SMCs within the matrigel were stained with DAPI for nucleus staining, and the immigrated SMCs in the bottom chamber were observed under the bright field. The images were captured and then analyzed using ImageJ software (National Institutes of Healthcare, Bethesda, USA) for fitting the boundary of the cells, as well as quantification of angle orientation and aspect ratio of SMCs. The cell orientation was measured from the best elliptical fit of the segmented cell body. All angles were normalized to be between 0° and 90°. The aspect ratio of SMCs was counted as the major and minor axis ratio.

Dysfunction of ECs and functional characterization of the HUVEC barrier was evaluated after atherogenic treatment using immunostaining and permeability test, respectively. Initially, Dil‐ox‐LDL was employed to observe the accumulation of hyperlipidemic agent after 24 and 48 h incubation using CLSM. Then, ROS secretion, stained by ROS probe (Keygen), and VE‐cad expression were analyzed for HUVECs in the artificial arterial wall to investigate the dysfunction and integrity of the HUVECs layer after 24 and 48 h incubation, respectively, along with the 2D plate culture as controls. Subsequently, ELISA (Mskbio, Wuhan, China) was performed following the manufacture instructions to qualify the recreations of specific proteins, that is, ox‐LDL, ROS, TG, and CH using the 3D AS model (ox‐LDL, 50 µg mL^−1^; TNF‐*α* at 2 ng mL^−1^, and IL‐1*β* at 2 ng mL^−1^), along with the 3D cultured cells incubated with lower hyperlipidemic and inflammatory factors (ox‐LDL, 25 µg mL^−1^; TNF‐*α* at 1 ng mL^−1^, and IL‐1*β* at 1 ng mL^−1^) and 2D plate culture as controls. The cells for ELISA assays were obtained and sonicated by ultrasonic emulsification (Scientz, Ningbo, China) for 1 min (ultrasonic treatment 5 s at 200 W, interval 5 s).

### Evaluation of Zinc Ions and Comparison between the Artificial Arterial Wall and 2D Petri Dish

After culture for determined time points within the culture medium complemented with zinc ions by dissolving ZnCl_2_ (Sinopharm, Beijing, China) in the culture medium, cell viability was investigated by the CCK‐8 assay, both in 2D petri dish culture and 3D engineered arterial wall. In brief, for 2D monolayer culture, 2500 HUVECs per well and 2500 SMCs per well were mixed and seeded into 96‐well microplates following incubation overnight. After hyperlipidemic and inflammatory stimulation, the cells were exposed to the culture medium with zinc ions following incubation for 24 h, along with the group without zinc ion treatment as the negative control. The dose‐response curve for zinc ions was obtained with concentrations ranging from 0 to 300 µm. After washing with PBS twice, CCK‐8 working solution (10 µL) was added to each well and incubated with fresh culture medium (100 µL) for 4 h. The optical densities at 450 nm of the supernatants were measured using a microplate reader (Thermo Fisher) with at least six parallel groups. In addition, 3D cell cultures were also measured based on the engineered arterial wall for cell viability investigations.

Subsequently, flow cytometry (Novocyte Quanteon, BD bioscience, Franklin Lakes, New Jersey) was then conducted for the analysis of the cell cycle using DAPI and apoptosis using annexin V‐fluorescein isothiocyanate/propidium iodide (FITC/PI), respectively. Briefly, cells were resuspended by staining with the working solution and catered to flow cytometry at 405 nm for DAPI and 488/552 nm for FITC/PI excitation wavelength, respectively. The cell cycle analysis was carried out as 10 µL min^−1^ and stopped at 20 000 events. Specifically, to reobtain cells within GelMA hydrogel for proliferation investigation, the sample was soaked in the GelMA lyase at 37 °C for 1 h and centrifuged to obtain cells.

### Evaluation of the Immune Response of MCs after Treatment with Zinc Ions

To explore the MC polarization after incubation with ox‐LDL/TNF‐*α*/IL‐1*β*, following treatment with the zinc ions at various concentrations (20.0 and 116.2 µm), the various MC markers, including genetic markers associated with cellular polarization including IL‐6 and CD86 for M1 markers, and IL‐10 and CD206 for M2 markers, were investigated using ELISA, immunofluorescence, and flow cytometry, respectively. First, ELISA was performed following the instructions of the manufacturer to qualify the secretion of these specific proteins. Then, the expression of CD86 and CD206 was stained by CD86 and CD206 polyclonal antibody (1:200 dilutions in PBS, Invitrogen, Carlsbad, USA), respectively, following incubation with secondary antibody (1:200 dilutions in PBS, Invitrogen) and DAPI working solution. The images were captured using CSLM and analyzed by ImageJ.

Further, cells analyzed by flow cytometry were stained with F4/80‐FITC (1:200 dilutions in PBS) and CD16/32‐PEcy7 (1:200 dilutions in PBS) for 30 min at 4 °C, following fixation and permeabilization, and stained with CD206‐APC for 30 min at 4 °C. The matching control PEcy7 and APC lgG isotype controls were employed, and cells were finally washed and resuspended in the staining buffer. M1 MCs were identified as F4/80+ and CD16/32+ cell surface markers, whereas M2 MCs were identified as F4/80+ and CD206+, and the samples were assessed as the flow rate of 10 µL min^−1^ and stopped at 20 000 events. All reagents for the flow cytometry were purchased from Invitrogen (Carlsbad, USA).

### Proteomic Analysis

The cells were collected, and the experiments were carried out using TMT (Thermo Fisher) approach in triplicates for each group. The total proteins were extracted and enzymatic desalination using XBridge peptide BEH Column 18 (5 µm, Waters Corporation, Framingham, USA). TMT reagents were added to each sample, and ammonia (Wako Pure Chemical Industries Ltd., Osaka, Japan) was added to terminate the reactions. The samples were then lyophilized overnight and subject to liquid chromatography‐tandem mass spectrometer (LC‐MS/MS, L‐3000, Rigol, Beijing, China). Quantification and normalization of protein spots were performed using Proteome Discoverer 2.4 (Thermo Fisher) and Homo Sapient Swiss‐Prot database. The GO was performed, and the identified proteins of each group of samples in the experiment were uploaded to the KEGG (http://www.genome.jp/kegg) website, and the map results of all pathways were obtained.

### Statistical Analysis

Statistical significance and one‐way analysis of variance were performed using GraphPad Prism (Version 9.0, GraphPad Software, Inc., La Jolly, USA) and determined at the level of *P* < 0.05. All experimental data of at least six parallel samples were presented as mean ± standard deviations eliminating maximum and minimum values in each group.

## Conflict of Interest

The authors declare no conflict of interest.

## Supporting information

Supporting Information

## Data Availability

The data that support the findings of this study are available from the corresponding author upon reasonable request.
