# Supplementary material for: Revealing the Role of Zinc Ions in Atherosclerosis Therapy via an Engineered Three‐Dimensional Pathological Model
Source: Adv Sci (Weinh). 2023 Apr 24;10(18):2300475. doi: 10.1002/advs.202300475 (PMC10288231; doi:10.1002/advs.202300475)
Supplement: Supplementary file 1 — Supporting Information [file ADVS-10-2300475-s001.pdf]

ADVANCED SCIENCE
Open Access

Supporting Information

for *Adv. Sci.*, DOI 10.1002/advs.202300475

Revealing the Role of Zinc Ions in Atherosclerosis Therapy via an Engineered Three-Dimensional Pathological Model

Ying Wang, Nan Huang and Zhilu Yang**

Supporting information

Revealing the role of zinc ions in atherosclerosis therapy *via* an engineered three-dimensional pathological model

Ying Wang, Nan Huang, Zhilu Yang**

Y. Wang, Prof. N. Huang, Prof. Z. Yang
The Tenth Affiliated Hospital of Southern Medical University
Dongguan, 523059, P. R. China
Email: wangying277@outlook.com; zhiluyang1029@smu.edu.cn

Y. Wang, Prof. Z. Yang
Guangdong Provincial Key Laboratory of Cardiac Function and Microcirculation
Guangzhou, 510080, P. R. China

Prof. Z. Yang
Department of Cardiology
Third People's Hospital of Chengdu Affiliated to Southwest Jiaotong University
Chengdu, 610031, P. R. China

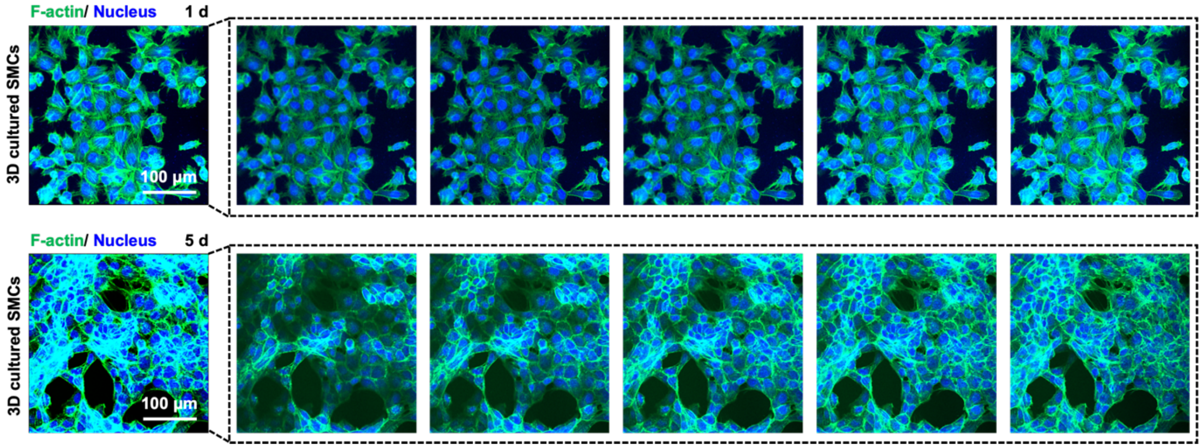

Figure S1. Overlayed and individual layer-by-layer fluorescence microscopy images for comprehending visualization of the 3D cultured SMCs and the coalignment of actin filaments of SMCs.

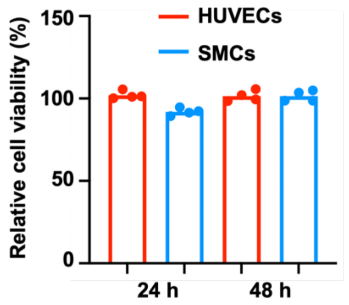

Figure S2. Cytotoxicity test *in vitro* showing the viabilities of individual HUVECs and SMCs after incubation with the mixture of of ox-LDL (50 μg mL^{-1}), TNF-α (2 ng mL^{-1}), and IL-1β (2 ng mL^{-1}) at different exposure times (24 and 48 h).

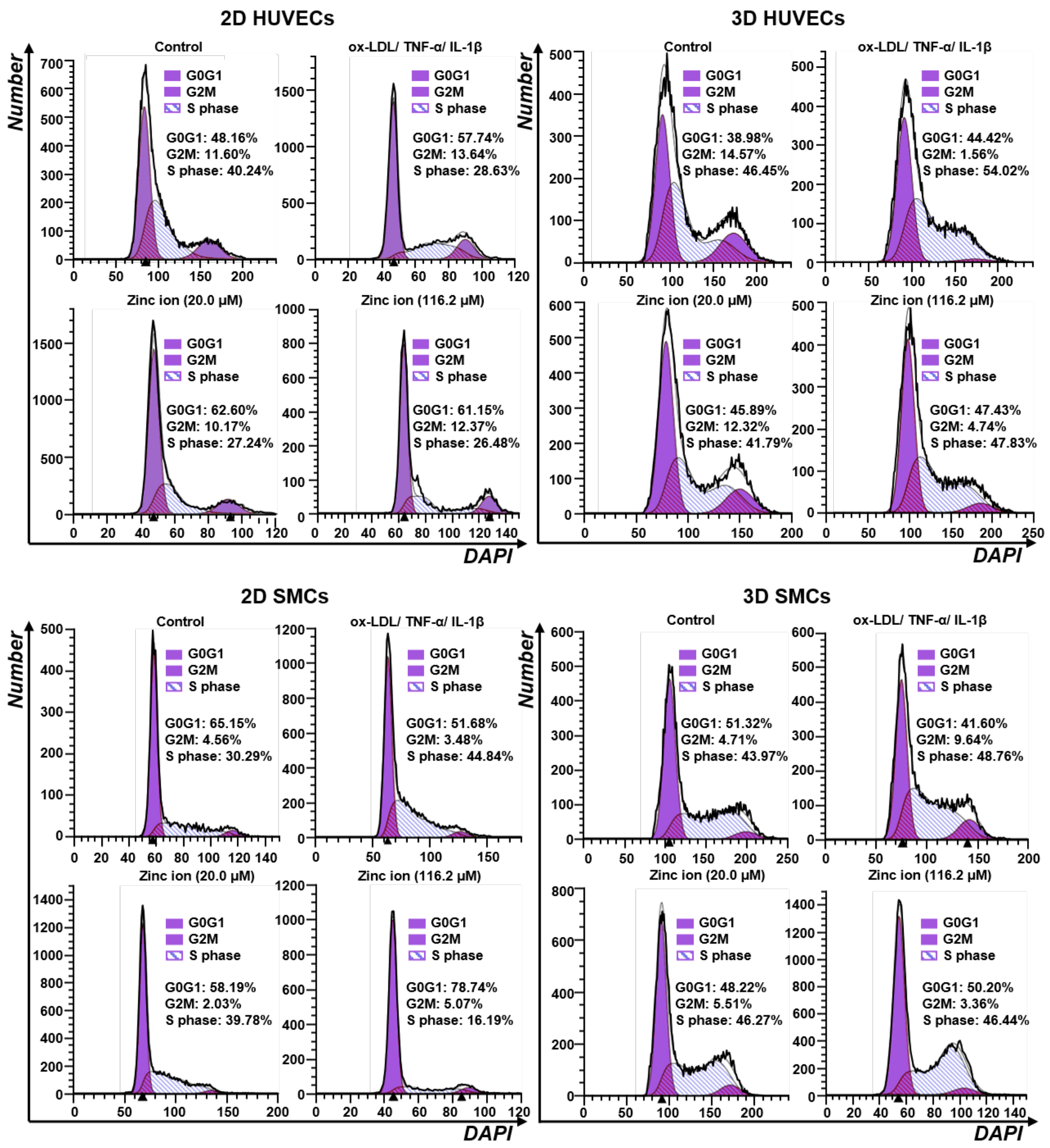

Figure S3. Cell cycle distinctions in 2D- and 3D-cultured HUVECs and SMCs, separately. The cells were stimulated by ox-LDL/ TNF-α/ IL-1β, following incubation with zinc ions at the concentration of 20.0 and 116.2 μM, respectively.

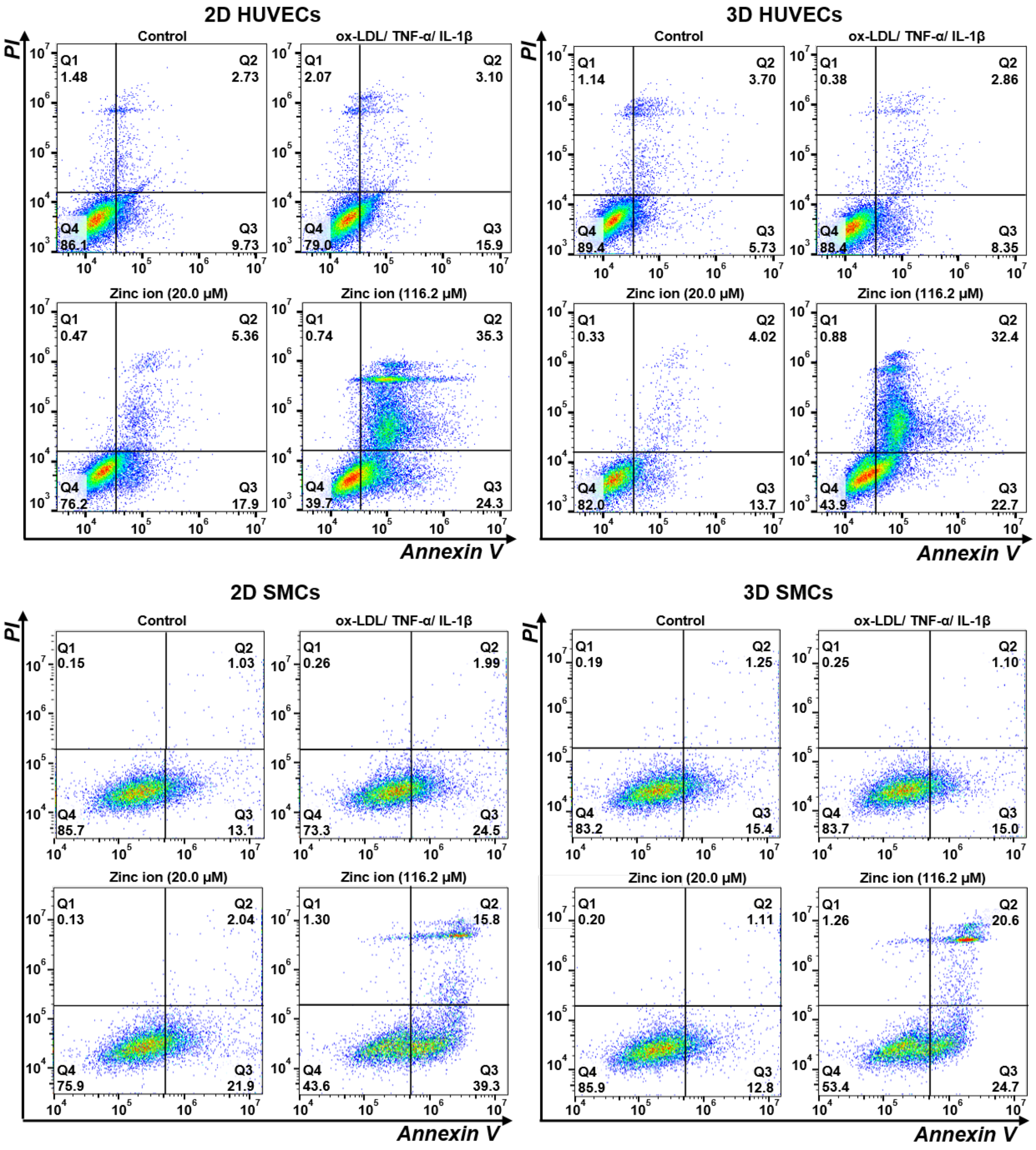

Figure S4. Cell apoptosis evaluations in 2D- and 3D-cultured HUVECs and SMCs, separately. The cells were stimulated by ox-LDL/ TNF-α/ IL-1β, following incubation with zinc ions at the concentration of 20.0 and 116.2 μM, respectively.

Table S1. KEGG pathway analysis and comparison (Control *vs* 3D AS model).

Term ID	Term	Gene Symbol
hsa04064	NF-kappa B signaling pathway	BCL2L1, PLAU, CD40, NFKB2, ICAM1, TRAF1
hsa05144	Malaria	SDC1, CD40, ICAM1
hsa05166	Human T-cell leukemia virus 1 infection	BCL2L1, CD40, HLA-B, JUN, NFKB2, ICAM1, FOSL1
hsa05202	Transcriptional misregulation in cancer	BCL2L1, PLAU, CD40, CEBPB, TRAF1
hsa05143	African trypanosomiasis	ICAM1, APOA1, APOL1
hsa05169	Epstein-Barr virus infection	CD44, CD40, CDK6, HLA-B, JUN, NFKB2, ICAM1
hsa04514	Cell adhesion molecules (CAMs)	SDC1, CD40, HLA-B, ICAM1
hsa05150	Staphylococcus aureus infection	KRT17, HLA-B, ICAM1
hsa04977	Vitamin digestion and absorption	APOB, APOA1
hsa04975	Fat digestion and absorption	APOB, APOA1
hsa04979	Cholesterol metabolism	APOC3, APOB, APOA1
hsa05323	Rheumatoid arthritis	HLA-B, JUN, ICAM1
hsa05310	Asthma	CD40, HLA-B
hsa04668	TNF signaling pathway	JUN, ICAM1, CEBPB, TRAF1
hsa05330	Allograft rejection	CD40, HLA-B
hsa04672	Intestinal immune network for IgA production	CD40, HLA-B
hsa05320	Autoimmune thyroid disease	CD40, HLA-B
hsa05222	Small cell lung cancer	COL4A1, BCL2L1, CDK6, TRAF1

Table S2. KEGG pathway analysis and comparison (3D AS model *vs* 3D AS model with zinc ion treatment at the concentration of 20 μM).

Term ID	Term	Gene Symbol
hsa04978	Mineral absorption	SLC30A1, HMOX1
hsa04610	Complement and coagulation cascades	C9, F5
hsa05150	Staphylococcus aureus infection	KRT17, HLA-C
hsa05322	Systemic lupus erythematosus	C9, HLA-C
hsa00532	Glycosaminoglycan biosynthesis - chondroitin sulfate / dermatan sulfate	CHST7
hsa04137	Mitophagy - animal	BNIP3, CALCOCO2
hsa05310	Asthma	HLA-C
hsa05332	Graft-versus-host disease	HLA-C
hsa05222	Small cell lung cancer	CKS1B, TRAF1
hsa04672	Intestinal immune network for IgA production	HLA-C
hsa05320	Autoimmune thyroid disease	HLA-C
hsa04940	Type I diabetes mellitus	HLA-C
hsa05330	Allograft rejection	HLA-C
hsa05200	Pathways in cancer	CKS1B, VHL, HMOX1, TRAF1
hsa04152	AMPK signaling pathway	SCD, EEF2K
hsa04066	HIF-1 signaling pathway	VHL, HMOX1
hsa01040	Biosynthesis of unsaturated fatty acids	SCD
hsa05164	Influenza A	HLA-C, CALCOCO2

Table S3. KEGG pathway analysis and comparison (3D AS model *vs* 3D AS model with zinc ion treatment at the concentration of 116.2 μM)

Term ID	Term	Gene Symbol
hsa05310	Asthma	HLA-G, CD40, HLA-C, HLA-E
hsa04672	Intestinal immune network for IgA production	HLA-G, CD40, HLA-C, HLA-E
hsa05320	Autoimmune thyroid disease	HLA-G, CD40, HLA-C, HLA-E
hsa05330	Allograft rejection	HLA-G, CD40, HLA-C, HLA-E
hsa05145	Toxoplasmosis	LDLR, NFKBIA, HLA-G, CD40, HSPA1B, LAMB3, HLA-C, HSPA6, HLA-E
hsa05219	Bladder cancer	DAPK3, RASSF1, CDKN1A, MYC, HBEGF
hsa04060	Cytokine-cytokine receptor interaction	GDF15, CD40, OSMR, TNFSF9
hsa04514	Cell adhesion molecules (CAMs)	ALCAM, HLA-G, MPZL1, CD40, HLA-C, HLA-E
hsa05332	Graft-versus-host disease	HLA-G, HLA-C, HLA-E
hsa04612	Antigen processing and presentation	CALR, HLA-G, HSPA1B, HLA-C, HSPA6, HLA-E
hsa04940	Type I diabetes mellitus	HLA-G, HLA-C, HLA-E
hsa04137	Mitophagy - animal	GABARAPL2, AMBRA1, TAX1BP1, GABARAP, BNIP3, HIF1A, CALCOCO2
hsa04640	Hematopoietic cell lineage	CD59, HLA-G, HLA-C, HLA-E
hsa04610	Complement and coagulation cascades	CD59, PROCR, F5
hsa05323	Rheumatoid arthritis	HLA-G, HLA-C, TCIRG1, HLA-E
hsa04979	Cholesterol metabolism	NPC2, LDLR, APOC3, APOB
hsa05150	Staphylococcus aureus infection	HLA-G, HLA-C, HLA-E
hsa05310	Asthma	HLA-G, CD40, HLA-C, HLA-E
